# Genetic variation in aryl *N*-acetyltransferase results in significant differences in the pharmacokinetic and safety profiles of amifampridine (3,4-diaminopyridine) phosphate

**DOI:** 10.1002/prp2.99

**Published:** 2014-12-09

**Authors:** Peter E Haroldsen, Marvin R Garovoy, Donald G Musson, Huiyu Zhou, Laurie Tsuruda, Boyd Hanson, Charles A O’Neill

**Affiliations:** BioMarin Pharmaceutical Inc.Novato, California, 94949

**Keywords:** 3,4-DAP, *N*-acetyltransferase, pharmacokinetics, NAT2 genotyping, Lambert–Eaton myasthenic syndrome

## Abstract

The clinical use of amifampridine phosphate for neuromuscular junction disorders is increasing. The metabolism of amifampridine occurs via polymorphic aryl *N*-acetyltransferase (NAT), yet its pharmacokinetic (PK) and safety profiles, as influenced by this enzyme system, have not been investigated. The objective of this study was to assess the effect of NAT phenotype and genotype on the PK and safety profiles of amifampridine in healthy volunteers (*N *=* *26). A caffeine challenge test and NAT2 genotyping were used to delineate subjects into slow and fast acetylators for PK and tolerability assessment of single, escalating doses of amifampridine (up to 30 mg) and in multiple daily doses (20 mg QID) of amifampridine. The results showed that fast acetylator phenotypes displayed significantly lower *C*_max_, AUC, and shorter *t*_1/2_ for amifampridine than slow acetylators. Plasma concentrations of the *N*-acetyl metabolite were approximately twofold higher in fast acetylators. Gender differences were not observed. Single doses of amifampridine demonstrated dose linear PKs. Amifampridine achieved steady state plasma levels within 1 day of dosing four times daily. No accumulation or time-dependent changes in amifampridine PK parameters occurred. Overall, slow acetylators reported 73 drug-related treatment-emergent adverse events versus 6 in fast acetylators. Variations in polymorphic NAT corresponding with fast and slow acetylator phenotypes significantly affects the PK and safety profiles of amifampridine.

## Introduction

Lambert–Eaton Myasthenic Syndrome (LEMS) is a rare and debilitating neuromuscular disorder characterized by the production of IgG autoantibodies that are directed against type P/Q voltage-gated calcium channels located on presynaptic neuronal membranes at the neuromuscular junction (Lambert et al. 1956; Augustine 1990; Verschuuren et al. 2006). As a result, the entry of calcium into neurons is impaired, inhibiting the normal release of acetylcholine and resulting in the loss of neuromuscular transmission.

Clinically, LEMS is characterized by proximal muscle weakness and fatigability, hyporeflexia, or areflexia, and symptoms of autonomic dysfunction such as impotence, dry mouth, and constipation (Lambert et al. 1956; Verschuuren et al. 2006). Other symptoms may include paresthesias, diplopia, and orthostatic hypotension (Wirtz et al. 2002). Proximal muscle weakness can compromise simple tasks such as walking and climbing stairs. The neuromuscular symptoms in patients with LEMS typically develop after 40 years of age with a peak incidence between 50 and 70 years of age (O’Neill et al. 1988; Wirtz et al. 2002). Although the exact prevalence of LEMS in the general population is unknown, it has been estimated to affect approximately 1 in 100,000 people (Sanders 2003).

Amifampridine (3,4-diaminopyridine; 3,4-DAP) is a nonspecific voltage-dependent potassium channel blocker (Thomsen and Wilson 1983; Maddison et al. 1998). Amifampridine prolongs presynaptic cell membrane depolarization by blocking potassium channels and facilitating the transport of calcium into nerve endings, thereby increasing the release of acetylcholine and improving neuromuscular transmission (Motomura et al. 1997). Amifampridine phosphate is approved in the European Union for the symptomatic treatment of LEMS (Firdapse® Tablets; BioMarin Europe Ltd. London), where 3,4-DAP is recommended as first-line therapy by the European Federation of Neurological Societies (Skeie et al. 2006, 2010). Previous clinical trials evaluated the safety and efficacy of amifampridine (Quartel et al. 2010), yet were not conducted with a prior knowledge of its metabolic disposition and impact of the latter on clinical outcomes.

During bioavailability studies in healthy volunteers, it was discovered that amifampridine undergoes 3-*N*-acetylation to form a single major circulating inactive metabolite that subsequently undergoes renal elimination (unpublished data on file, BioMarin). Approximately 80% of an orally administered dose of amifampridine is metabolized and excreted in this manner. *N*-acetylation of arylamines occurs by polymorphic arylamine *N*-acetyltransferase types 1 (NAT1) and 2 (NAT2). The acetylation rate of many arylamines is known to be affected by NAT2 polymorphism resulting in “slow” and “fast” acetylator phenotypes (Parkinson et al. 2010). In the general population, the NAT2 slow acetylator phenotype is more common among European and North American natives, including Caucasians and African populations (40–70%) than those with Pacific Asian ethnic backgrounds (10–30% among Japanese, Chinese, Korean, and Thai) (Meyer and Zanger 1997).

Slow acetylators tend to accumulate drug metabolized by NAT to higher levels that can influence their efficacy, side effect profile, and toxicity (Fukino et al. 2008; Jetter et al. 2009). Thus, we hypothesized that variations in amifampridine plasma pharmacokinetic (PK) parameters (unpublished data on file, BioMarin) and side effects observed in previous human studies (Wirtz et al. 2010) and identical to those observed in this study, are related to NAT polymorphism. Here, we discovered and studied the correlation of the PK and side effect profile of amifampridine and major metabolite 3-*N*-acetyl amifampridine within a defined individual acetylation phenotype and NAT2 gene polymorphism genotype. The findings of the present study should provide for a better understanding of the relationship between amifampridine administration and occurrence of adverse events (AEs) in the greater population of individuals taking the drug for LEMS or other indications.

## Methods

### Study objectives

This was a Phase 1, open-label study to evaluate the safety, tolerability, and PKs of single and multiple oral doses of amifampridine phosphate in healthy male and female subjects covering the range of recommended prescribed dose levels (BioMarin clinical trial FIR-001; European Union drug regulating authorities clinical trials [EudraCT[ number: 2011-000596-13). The objectives of the study were to assess the safety and tolerability, to assess the PK profile of amifampridine and the major 3-*N*-acetyl metabolite in defined slow and fast acetylator phenotypes, and to correlate the PK profile of amifampridine and its major metabolite with phenotypic acetylation activity and NAT2 gene polymorphism genotype after single and repeat doses of amifampridine phosphate.

### Acetylation phenotyping: caffeine challenge test

To delineate enrolled subjects into groups of slow and fast acetylators (1:1), the acetylation phenotype of each subject was characterized using the caffeine challenge test 1 week prior to receiving study drug (Schneider et al. 2003). A single urine sample was analyzed from a pooled 6-h collection obtained from each subject following a 150 mg oral dose of caffeine. The concentration of 5-acetylamino-6-formylamino-3-methyl uracil (AFMU), acetylamino-6-amino-3-methyluracil (AAMU), 1-methylxanthine (1X), and 1-methylurate (1U) were determined by liquid chromatography-mass spectrometry/mass spectrometry (LC-MS/MS, Schneider et al. 2003). From the measured concentrations, the overall acetylation rate (NAT2_activity_) was calculated according to the method of Schneider et al. (2003) as:

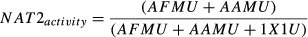
where AFMU and AAMU are *N*-acetylated products of caffeine metabolism and 1X and 1U are metabolites formed by another route. Slow acetylators were defined as persons with *N*-acetylated caffeine metabolite ratios <0.2, while fast acetylators had *N*-acetylated caffeine metabolite ratios >0.3. Subjects with intermediate caffeine metabolite ratios ≥0.2, but ≤0.3, did not participate in the study. Subjects with caffeine metabolite ratios ≤0.06 were considered ultraslow acetylators and were not enrolled due to safety concerns and possible drug accumulation.

### NAT2 polymorphism genotyping

Using blood samples obtained the day before receiving study drug, all subjects underwent genotyping for variant alleles on the NAT2 domain of chromosome 8 after the study was completed. Genomic DNA was isolated from Ethylenediaminetetraacetic acid (EDTA) treated blood using standard methods (Cascorbi et al. 1995). NAT2 genotypes were determined using polymerase chain reaction with allelic discrimination (Applied Biosystems® StepOnePlus™ Real-Time PCR System and TaqMan® SNP Genotyping Assay; Life Technologies Corporation, Grand Island, NY). The identified NAT2 variations (NAT*4, *5A, *5B, *5C, *6A, *7B, *13, and *14B) were detected by 2 probes (C282T and T341C). These probes identify the linked allelic variations at positions 282 and 341 on chromosome 8, which together represent >95% of the known NAT2 variants (Cascorbi et al. 1995). As an autosomal recessive trait, only individuals with two variant alleles result in the slow acetylator phenotype. Thus, the two potential classes of acetylator activity were one or two fast alleles producing fast acetylators and no fast alleles producing slow acetylators. A summary of possible NAT2 gene polymorphisms is provided in Table S1 and the actual genotypes of enrolled subjects are summarized in Table 1.

**Table 1:** Summary of NAT2 genotypes of enrolled subjects based on identified phenotype

	Fast acetylators			Slow acetylators		
	WT/WT	HE/WT	WT/HE	HE/HE	MU/WT	WT/MU
Part 1^1^, *N* (%)	–	3 (25)	3 (25)	1 (8)	3 (25)	2 (17)
Part 2^2^, Group 1, *N* (%)	–	2 (50)	–	2 (50)		–
Part 2^3^, Group 2, *N* (%)	2 (20)	1 (10)	2 (20)	3 (30)	2 (20)	–

Genotype: WT, wild type; HE, heterozygote; MU, mutant.

1 Treatment was 5–30 mg single oral dose.

2 Treatment was 20 mg oral amifampridine doses QID for 1 day.

3 Treatment was 20 mg oral amifampridine doses QID for 3 days followed by a single dose on day 4.

### Subjects

Study participants included healthy men and women aged 18–55 years, with a body mass index (BMI) of 18–30 kg/m^2^. Each subject expressed their willingness to follow the study protocol requirements of abstaining from alcohol, methylxanthine-containing beverages or food (coffee, tea, cola, chocolate), poppy seeds, and grapefruit juice during the study or any medication or herbal remedy known to alter drug absorption or metabolism within 30 days of receiving the first dose of study drug.

### Study drug

The study drug was provided as Firdapse® (amifampridine phosphate) Tablets (BioMarin Europe Ltd., London, UK). Each tablet contained 18.98 mg of amifampridine phosphate, which is equivalent to 10 mg of amifampridine free base. Presently, the recommended total daily dose range is 15–60 mg taken in divided doses with no single dose to exceed 20 mg (Firdapse® Tablets Prescribing Information 2013). Without knowledge of a patient’s acetylator status, dose administration is initiated at the 15 mg/day level and slowly increased in 5 mg increments every 4–5 days to a maximum of 60 mg per day, depending on the patient’s tolerability and efficacy outcome (Firdapse® Tablets Prescribing Information 2013). The present study evaluated and exceeded the present clinically relevant dose levels.

### Study design

#### Part 1

The 12 subjects participating in Part 1 consisted of 2 groups, each with six fast or six slow acetylators. Over a 4-day period, single ascending amifampridine doses of 5 mg (1/2 tablet), 10 mg (1 tablet), 20 mg (2 tablets), and 30 mg (3 tablets) were administered to each subject at about 08:00 h and within 30 min following a standardized breakfast given to each subject at all dose levels. At the highest dose level of 30 mg, two subjects from each group first received a 30 mg dose of amifampridine and served as sentinel subjects for AE evaluation prior to continuing dose administration to the additional 4 subjects in each group.

#### Part 2

The 14 subjects participating in Part 2 consisted of seven fast and seven slow acetylators. Two subjects from each group first received a 1-day QID multiple dosing regimen and served as sentinel subjects for AEs. Subjects in this sentinel Group 1 of Part 2 were administered four doses of amifampridine 20 mg (2 tablets) at 4-h dosing intervals (8:00, 12:00, 16:00 and 20:00 h) within 30 min following a standardized meal or snack (Food and Drug Administration 2010). All remaining subjects in Group 2 of Part 2 were administered four doses of amifampridine 20 mg (2 tablets) at 4-h dosing intervals (8:00, 12:00, 16:00, and 20:00 h) for 3 days and a final morning dose at 08:00 h on Day 4. Each dose was administered within 30 min of a standard meal or snack.

### Amifampridine and 3-*N*-acetyl amifampridine sampling

#### Part 1

Blood samples for plasma amifampridine and 3-*N*-acetyl amifampridine analysis were obtained prior to receiving amifampridine and at 10, 20, 30, 45, 60, 75, 90 min and 2, 4, 6, 8, 10, 12, 16, and 24 h afterward.

#### Part 2, Group 1

Blood samples were obtained prior to receiving the first and third doses of amifampridine and at 10, 20, 30, 45, 60, 75, 90 min and 2, 3, and 4 h afterward. Blood samples were also obtained 30, 60, 90 min and 2 and 4 h after receiving the second and fourth doses.

#### Part 2, Group 2

On Days 1 and 3, blood samples were obtained prior to receiving the first and third doses of amifampridine and at 10, 20, 30, 45, 60, 75, 90 min and 2, 3, and 4 h afterward. Blood samples were also obtained 30, 60, 90 min and 2 and 4 h after the second and fourth doses on Days 1 and 3. On Day 2, single trough samples were collected in the morning and afternoon prior to the first and third doses. On Day 4, blood samples were obtained prior to receiving amifampridine and at 10, 20, 30, 45, 60, 75, 90 min and 2, 4, 6, 8, 10, 12, 16, and 24 h afterward.

### Amifampridine and 3-*N*-acetyl amifampridine analysis

Plasma concentrations of amifampridine and its 3-*N*-acetyl metabolite were determined using protein precipitation followed by high-performance liquid chromatography (HPLC) and tandem mass spectrometric detection (Bioanalytical Sciences, BioMarin Pharmaceutical, Inc., Novato, CA). Briefly, 25 *μ*L of plasma sample was mixed with 300 *μ*L of ACN containing the internal standards and 0.1% formic acid. After centrifugation, 200 *μ*L of supernatant was transferred to a clean 96-well plate and injected into the LC/MS/MS system. The method employed individual stable isotope-labeled internal standards for amifampridine ([^2^H_3_[-3, 4- diaminopyridine) and 3-*N-*acetyl amifampridine ([^2^H_3_[-3-*N*-acetyl). The HPLC system consisted of two Shimadzu 20-AD pumps (Shimadzu Scientific Instruments, Columbia, MD, USA) operated in a gradient flow (90% organic mobile phase B to 90% aqueous mobile phase A); [A = 20mM NH4formate/0.1% formic acid; B= acetonitrile/isopropanol (90/10) + 0.1 % formic acid) with an Atlantis HILIC silica column (3 × 50 mm, 3 *μ*m particle size). The entire eluent was transferred directly to a 4000 QTRAP mass spectrometer (AB Sciex, Foster City, CA) operated in a positive ESI mode with tandem quadrupole mass filtering. The settings to monitor each analyte consisted of a characteristic protonated precursor ion (M+H^+^) to product ion mass transition as follows: amifampridine (110→93 Da), ^2^H_3_-amifampridine (113→96 Da), 3-*N*-acetyl amifampridine (152→110 Da), and 3-*N*-^2^H_3_-acetyl amifampridine (155→111 Da). Each precursor-product ion transition was optimized to a particular collision energy (30–40 eV) in the quadrupole collision cell using nitrogen as collision gas. The lower limit of quantitation for amifampridine was 0.5 ng/mL and 1 ng/mL for the metabolite. Concentration results were calculated using a linear calibration curve of drug-to-internal standard or metabolite-to-internal standard peak area ratios and calibration curves generated using 1/X^2^ weighted linear least squares regression. The assay performance was monitored daily with QC samples using blank plasma samples spiked with reference standards as follows for Amifampridine: Low QC [2 ng/mL; mean accuracy 102% (*N* = 58) and range 84.0–115% nominal[, Mid QC [50 ng/mL; mean accuracy 104% (*N* = 58) and range 91.0–115% nominal[, High QC [400 ng/mL; mean accuracy 99% (*N* = 58) and range 83.3–109% nominal[ and for 3-*N*-acetyl amifampridine metabolite: Low QC [4 ng/mL; mean accuracy 101% (*N* = 54) and range 79.8–110% nominal[, Mid QC [100 ng/mL; mean accuracy 102% (*N* = 54) and range 88.3–115% nominal[, High QC [800 ng/mL; mean accuracy 97.1% (*N* = 54) and range 82.1–110% nominal[.

### Pharmacokinetic analysis

Plasma concentration-time data were used to calculate PK parameters for amifampridine and the 3-*N*-acetyl metabolite using noncompartmental analysis (Phoenix WinNonlin 6.1; Pharsight Corporation, Cary, NC). The values for *C*_max_ and *T*_max_ were obtained directly from plasma-time concentration data by inspection without interpolation. The values for AUC_0-*t*_ were calculated by the linear trapezoidal rule. The AUC_0-4h_ values for each multiple dosing interval in Part 2 were calculated using a 0–4 h reference time interval (not clock time) for each of the four doses on Days 1 and 3. The apparent *t*½ was calculated by 0.693/*λz*, where the terminal elimination rate constant *λz* was determined by log-linear regression of the terminal plasma concentrations. The AUC_0-∞_ was calculated by AUC_0-*t*_ + C*t*/*λz*, where C*t* is the last measurable plasma concentration for measured drug or metabolite. The apparent oral clearance CL/*F* was calculated by dose/AUC_0-∞_, where *F* represents the unknown fraction of absorbed drug. The apparent oral volume of distribution *V*_dz_/*F* was calculated based on the terminal elimination rate constant by (CL/F) · 1/*λz*. The apparent oral volume of distribution at steady state *V*_dss_/*F* was calculated as MRT_0-∞_ · CL/*F*, where MRT_0-∞_ is the mean residence time extrapolated from time 0 to infinity.

### Safety and tolerability

The safety and tolerability profiles of amifampridine were assessed throughout the study by spontaneously reported AEs and those revealed to the investigator by inquiry. All AEs were assessed and coded by the investigator using the Medical Dictionary for Regulatory Activities (MedDRA, Version 13.1; Northrop Grumman Corporation, Falls Church, VA, USA). Additional safety measures included periodic physical examinations, vital signs, ECG (specifically, PR- and QT-interval, QRS-duration, QTc interval [Fridericia’s QTcF[ and heart rate), and EEG. Clinical laboratory measures included serum chemistry, hematology, urinalysis, serology, drug screen, thyroid hormones, and pregnancy tests.

### Statistical analysis

An analysis of variance model was performed on AUC_0-∞_, *t*½, *C*_max,_ and CL/F using statistical programming method PROC MIXED in SAS (version 9.1.3 or higher, SAS Institute Inc., Cary, NC). The parameters were natural logarithm transformed prior to analysis. The model included treatment (dose) and subgroup (acetylator type, gender, race, etc.) as fixed effects and subject as random effect (general model: log (PK) = treatment subgroup treatment·subgroup). From this model, the least squares means for each treatment by subgroup are represented, including the ratio of least squares geometric means and the corresponding 90% confidence intervals.

### Ethics

This study was conducted at a single research facility (Pharmaceutical Research Associates, Zuidlaren, The Netherlands). The protocol and informed consent forms were approved by an Independent Ethics Committee (Stichting Beoordeling Ethiek Bio-Medisch Onderzoek, Assen, The Netherlands). Each subject provided informed consent prior to participating in any study-related activities. The study was conducted in accordance with the principles of the Declaration of Helsinki and in compliance with the International Conference on Harmonisation E6 Guideline for Good Clinical Practice, and the European Union Clinical Trial Directive: Directive 2001/20/EC.

## Results

The enrolled subjects included men (*N *=* *18) and women (*N *=* *8) representing 13 slow and 13 fast acetylator phenotypes. The mean (SD) age of these subjects was 29 (11) years (range, 18–54 years) and their mean BMI was 23.8 (2.9) kg/m^2^ (range, 18.2–28.7 kg/m^2^). Subjects described themselves as White (*N *=* *23), American Indian or Alaska Native (*N *=* *1), White/African-American (*N *=* *1), and White/Asian (*N *=* *1).

### Part 1, single-dose pharmacokinetics

Single, ascending, oral doses of amifampridine resulted in dose-dependent increases in plasma amifampridine concentrations over the dose range of 5–30 mg in both slow and fast acetylator phenotypes; however, slow and fast acetylator phenotypes produced substantial differences in PK parameters for amifampridine.

**Figure 1:**
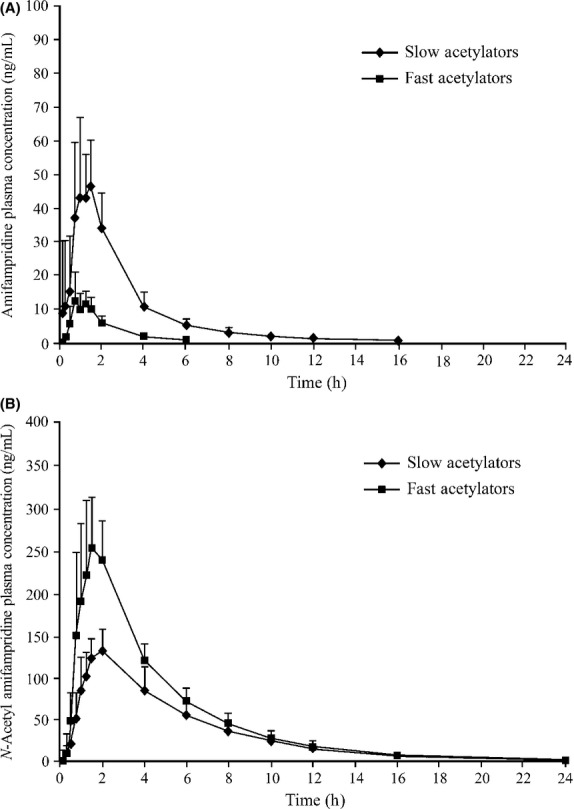
Mean plasma concentration-time profiles (+SD) for amifampridine and 3-*N*-acetyl amifampridine in subjects with slow and fast acetylator phenotypes following a single 20 mg oral amifampridine dose. (A) The mean amifampridine *C*_max_ values in slow acetylators (17.9–89.6 ng/mL) were 3.5- to 4.5-fold higher than fast acetylators (3.98–25.5 ng/mL). (B) The mean 3-*N*-acetyl amifampridine *C*_max_ values ranged from 82.3 to 350 ng/mL in fast acetylators versus 43.2–189 ng/mL in slow acetylators.

The mean *C*_max_ values in slow acetylators (17.9–89.6 ng/mL) were 3.5- to 4.5-fold higher than fast acetylators (3.98–25.5 ng/mL) (Table 2; Fig. 1A). The amifampridine *C*_max_ values increased in a dose-proportional manner (approximately 1:1) in response to the 6-fold increase in dose in slow (5.01-fold increase) and fast (6.41-fold increase) acetylator groups. For exposure based on *C*_max_ linear regression, analysis on mean *C*_max_ versus dose indicates linearity across the full range of doses tested (*R*^2^ > 0.990).

**Table 2:** Mean amifampridine and 3-*N*-acetyl amifampridine pharmacokinetic parameters in fast and slow acetylators after single oral doses

Amifampridine dose	5 mg		10 mg		20 mg		30 mg	
Acetylator phenotype (*N*)	Fast (6)	Slow (6)	Fast (6)	Slow (6)	Fast (6)	Slow (6)	Fast (6)	Slow (6)
Amifampridine mean PK parameters (SD)								
AUC_0-*t*_ (ng·h/mL)	2.89 (0.66)	30.1 (7.25)	9.55 (1.77)	66.3 (12.8)	24.7 (2.47)	142 (32.1)	43.5 (6.39)	230 (44.9)
AUC_0-∞_ (ng·h/mL)	3.57 (0.59)	32.1 (7.34)	11.1 (1.90)	68.9 (12.8)	26.2 (2.62)	146 (31.4)	45.2 (6.44)	234 (44.7)
*C*_max_ (ng/mL)	3.98 (1.71)	17.9 (4.43)	9.91 (5.28)	34.4 (21.6)	16.2 (4.56)	56.7 (16.1)	25.5 (7.17)	89.6 (9.05)
*T*_max_ (h)	0.75 (0.39)	0.83 (0.41)	0.81 (0.41)	1.14 (0.49)	1.04 (0.37)	1.07 (0.53)	0.810 (0.41)	1.29 (0.46)
*t*_1/2_ (h)	0.60 (0.30)	2.22 (0.86)	1.21 (0.28)	2.60 (0.69)	1.23 (0.31)	2.93 (0.59)	1.65 (0.63)	3.11 (0.57)
CL/F (L/h)^1^	1431 (234)	163 (37.4)	920 (155)	150 (32.1)	770 (67.5)	143 (32.3)	675 (98.5)	132 (20.5)
*V*_d*z*_/F (L)^1^	1254 (622)	509 (199)	1575 (343)	577 (252)	1363 (337)	607 (211)	1621 (703)	592 (146)
*V*_dss_/*F* (L)^1^	1763 (780)	434 (142)	1577(516)	459 (175)	1682 (365)	481(181)	1590 (374)	430 (79.9)
3-*N*-acetyl amifampridine mean PK parameters (SD)								
AUC_0-*t*_ (ng·h/mL)	286 (33.9)	205 (37.4)	609 (82.6)	422 (81.2)	1199 (120)	801 (128)	1687 (190)	1115 (185)
AUC_0-∞_ (ng·h/mL)	295 (33.0)	212 (35.6)	619 (83.5)	434 (79.6)	1213 (119)	818 (130)	1706 (190)	1140 (185)
*C*_max_ (ng/mL)	82.3 (21.8)	43.2 (14.5)	162 (56.2)	80.6 (12.7)	268 (57.5)	138 (21.1)	350 (40.5)	189 (31.8)
*T*_max_ (h)	1.13 (0.57)	1.21 (0.40)	1.25 (0.45)	1.50 (0.63)	1.58 (0.47)	1.75 (0.42)	1.50 (0.42)	1.67 (0.41)
*t*_1/2_ (h)	3.06 (0.57)	3.72 (1.11)	3.78 (1.25)	4.29 (1.21)	3.63 (1.01)	4.31 (0.63)	3.63 (0.64)	4.35 (0.50)

1 These parameters were calculated in milliliters and converted to liters.

The mean amifampridine AUC_0-*t*_ values ranged from 2.89 to 43.5 ng·h/mL in fast acetylators versus 30.1–230 ng·h/mL in slow acetylator phenotypes across the tested dose range. The mean AUC_0-*t*_ values for slow acetylators were approximately 5- to 10-fold higher than fast acetylators (Table 2) and increased in a greater than dose-proportional manner in slow (7.64-fold) and fast (15.1-fold) acetylator phenotypes over a sixfold increase in dose. For exposure based on AUC, linear regression analysis on mean AUC_0-∞_ versus dose indicates linearity (*R*^2^ > 0.995) across the full dose range tested.

**Figure 2:**
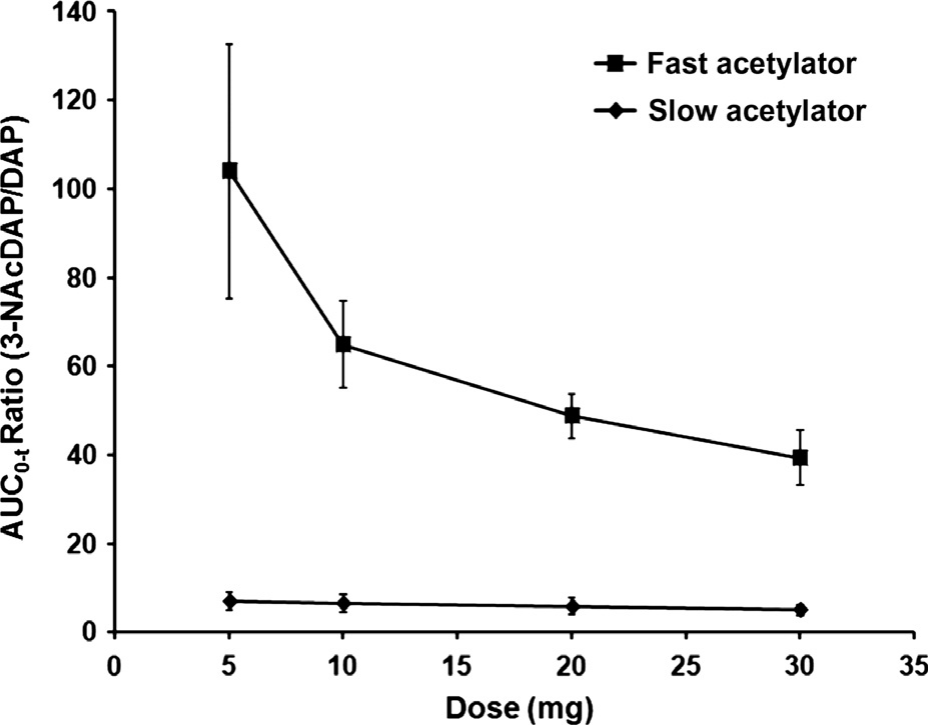
Plots of 3-*N*-acetyl amifampridine/amifampridine exposure ratios and dose in subjects with slow and fast acetylator phenotypes. Metabolite/amifampridine ratios for mean AUC_0-*t*_ values in the slow and fast acetylator NAT2 phenotypes. In fast acetylators, the ratios for mean AUC_0-t_ values ranged from 104- to 38.8-fold over the ascending dose range. In slow acetylators, the ratios for mean AUC_0-*t*_ values ranged from 7.07- to 4.85-fold.

The observed clearance (CL/F) for slow (2.2–2.7 L/min) and fast (11.3–23.9 L/min) acetylator phenotypes exceeded human hepatic blood flow (approximately 1.5 L/min), indicating the occurrence of extra-hepatic metabolism in combination with less than complete hepatic extraction. In addition, the observed clearance decreased in a dose-dependent manner in parallel with metabolite/drug exposure ratios (Fig. 2) for both phenotypes, implicating a saturable first-pass metabolism as a contributing component to the change in elimination with increasing dose. Plasma amifampridine exhibited biexponential elimination with mean terminal elimination half-lives ranging from 0.603 to 1.65 h in fast acetylators and 2.22–3.11 h in slow acetylators. Based on amifampridine acetylation phenotypes, the differences in the PK parameters *C*_max_, AUC, CL/F, and *t*_½_ were significant for all tested doses at the *P *<* *0.001 level (Table 3). There were no differences in *C*_max_, *t*_1/2,_ and AUC_0-4h_ between male (*N* = 3 fast, *N* = 4 slow) and females (*N* = 3 fast, *N* = 2 slow) in rapid or slow acetylator groups following single dosing in Part 1. No clear relationship between subject age and the amifampridine PK parameters or subject age and NAT caffeine metabolite ratio was observed in this limited set of subjects (*N* = 6 slow and *N* = 6 fast).

**Table 3:** Amifampridine pharmacokinetic parameters for fast and slow acetylator phenotypes

Amifampridine dose	Parameter	Geometric LS means ratio slow/fast (90% CI)	Significance
5 mg	*C*_max_	4.72 (3.21, 6.95)	*P *<* *0.0001
	AUC_0-∞_	8.84 (7.41, 10.6)	*P *<* *0.0001
	*t*_½_	3.85 (2.81, 5.29)	*P *<* *0.0001
	CL/F	0.113 (0.0947, 0.135)	*P *<* *0.0001
10 mg	*C*_max_	3.38 (2.29, 4.98)	*P *<* *0.0001
	AUC_0-∞_	6.14 (5.14, 7.33)	*P *<* *0.0001
	*t*_½_	2.13 (1.55, 2.93)	*P *=* *0.0004
	CL/F	0.163 (0.136, 0.195)	*P *<* *0.0001
20 mg	*C*_max_	3.45 (2.35, 5.09)	*P *<* *0.0001
	AUC_0-∞_	5.49 (4.62, 6.53)	*P *<* *0.0001
	*t*_½_	2.41 (1.78, 3.27)	*P *<* *0.0001
	CL/F	0.182 (0.153, 0.217)	*P *<* *0.0001
30 mg	*C*_max_	3.62 (2.46, 5.34)	*P *<* *0.0001
	AUC_0-∞_	5.14 (4.32, 6.11)	*P *<* *0.0001
	*t*_½_	1.96 (1.44, 2.66)	*P *=* *0.0008
	CL/F	0.195 (0.164, 0.231)	*P *<* *0.0001

Similar to the parent compound, plasma 3-*N*-acetyl amifampridine concentrations increased in a dose-dependent manner (Table 2; Fig. 1B) and exceeded amifampridine at all measured doses and time points in both acetylator groups. In contrast to amifampridine, however, 3-*N*-acetyl amifampridine plasma concentrations were consistently higher among fast acetylators. The mean *C*_max_ values ranged from 82.3 to 350 ng/mL in fast acetylators and 43.2–189 ng/mL in slow acetylators. With respect to AUC_0-*t*_, mean 3-*N*-acetyl amifampridine ranged from 286 to 1687 ng·h/mL in fast versus 205–1115 ng·h/mL in slow acetylator phenotypes. 3-*N*-acetyl amifampridine exhibited a monoexponential decay with mean terminal elimination half-life ranging from 3.06 to 3.78 h in fast acetylators versus 3.72–4.35 h in slow acetylator phenotypes (Table 2). The longer metabolite half-life in the slow acetylators is attributable to the longer duration of metabolite formation by the presence of higher and more persistent NAT2 amifampridine substrate levels.

The mean *C*_max_ and AUC values for 3-*N*-acetyl amifampridine were approximately 2-fold and approximately 1.5-fold higher, respectively, in the fast acetylator group. Similar to the parent compound, linear regression analysis of mean *C*_max_ and mean AUC_0-∞_ values versus dose indicated that the 3-*N*-acetyl metabolite exhibited linear plasma PK over the tested dose range (*R*^2^ > 0.980). Calculations of metabolite/amifampridine ratios for mean *C*_max_ and mean AUC_0-∞_ exposure parameters further demonstrated the differences between fast and slow acetylator phenotypes and genotypes (Fig. 2). Over the ascending dose range (5–30 mg), the ratios for mean *C*_max_ values ranged from 20.7-fold (5 mg) to 13.7-fold (30 mg) in fast and 2.41-fold (5 mg) to 2.11-fold (30 mg) in slow acetylators and the mean AUC_0-∞_ ratios ranged from 82.6-fold (5 mg) to 37.7-fold (30 mg) in fast acetylators and 6.60-fold (5 mg) to 4.87-fold (30 mg) in slow acetylators. The descending metabolite/amifampridine ratios with increasing oral dose implicate a saturable first-pass metabolism.

### Part 2, multiple dose pharmacokinetics

**Figure 3:**
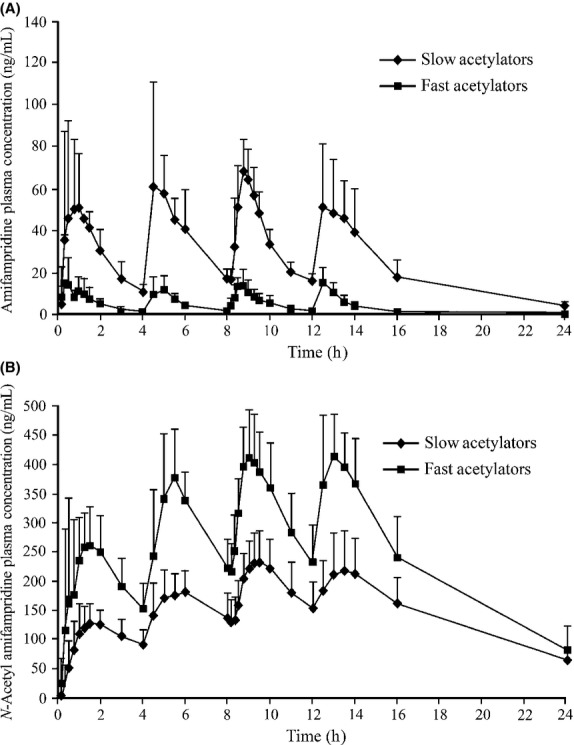
Mean plasma concentration-time profiles (+SD) for amifampridine and 3-*N*-acetyl amifampridine in subjects with slow and fast acetylator phenotypes after multiple 20 mg oral amifampridine doses (QID). (A) Amifampridine demonstrated rapid absorption, distribution, and plasma clearance during multiple dosing. Fast acetylators had consistently lower amifampridine plasma concentrations and systemic exposure than slow acetylators. (B) Rapid changes in 3-*N*-acetyl amifampridine plasma concentrations indicate rapid formation and elimination. Metabolite concentrations were approximately twofold higher in fast acetylators.

During multiple (QID) amifampridine dosing, observed changes in amifampridine plasma concentrations indicate that rapid absorption, distribution, and plasma clearance occurs with each dose (Fig. 3A). Similar to single dosing, rapid acetylators demonstrated consistently lower plasma amifampridine concentrations and systemic exposure than slow acetylators with multiple dosing. The mean amifampridine *C*_max_ values ranged from 13.3 to 24.4 ng/mL in rapid acetylators versus 67.1–97.1 ng/mL in slow acetylators (Table S2) across all PK sampling days. The mean AUC_0-4h_ values ranged from 22.5 to 28.5 ng·h/mL in fast acetylators and 115–168 ng·h/mL in slow acetylators across all PK sampling days. Similarly, mean daily exposure AUC_0-24h_ ranged from 97.9 to 111 ng·h/mL in fast acetylators and from 630 to 701 ng·h/mL in slow acetylators on Days 1 and 3, respectively, indicating an approximate sixfold difference in total systemic drug exposure between the acetylator phenotypes.

After administering the study drug four times daily for 3 days, the mean terminal elimination *t*_1/2_ for the final single dose of amifampridine on Day 4 was 1.95 h in rapid acetylators versus 3.24 h in slow acetylators, with a statistically significant difference at the *P *<* *0.05 level. Observed differences in PK parameters *C*_max_ and AUC_0-4h_ between rapid and slow acetylators were statistically significant at the *P *<* *0.002 level. Similar to Part 1, there were no differences in *C*_max_, *t*_1/2_, and AUC_0-4h_ between males and females in rapid or slow acetylator groups following multiple dosing in Part 2. No clear relationship between subject age and the amifampridine PK parameters or subject age and NAT caffeine metabolite ratio was observed in this limited set of subjects. Based on plasma *C*_min_ values, steady state plasma concentrations were achieved after dose 2 for amifampridine and after dose 3 for the 3-*N*-acetyl metabolite in both acetylator groups, indicating that steady state is reached within 1 day of an oral QID amifampridine dosing regimen.

Based on *C*_max_ ratio values obtained for Doses 1 and 4, negligible same-day accumulation of amifampridine occurs in rapid acetylators (0.875- to 1.10-fold) and little accumulation occurs in slow acetylators (1.08- to 1.48-fold). Although the mean interday *C*_max_ ratios were more equivocal with a range of values of 0.776- to 1.56-fold in rapid acetylators and 0.854- to 1.57-fold in slow acetylator groups, the data overall indicate that significant amifampridine accumulation did not occur after multiple dosing over the evaluated 4 days.

The AUC_0-4h_ ratio values for doses 1 and 4 also indicate same-day accumulation of amifampridine is negligible in rapid acetylators (1.03- to 1.07-fold increase) and low in slow acetylators (1.29- to 1.33-fold increase). The accumulation of amifampridine based on AUC_0-24h_ from Days 1 to 4 was negligible in both fast (1.02- to 1.15-fold) and slow acetylators (1.02- to 1.09-fold).

The plasma concentrations of the 3-*N*-acetyl metabolite also quickly increased and decreased with multiple dosing, indicating rapid formation and elimination (Fig. 3B). Exposure to the 3-*N*-acetyl metabolite was approximately 2-fold higher in the rapid acetylator group (PK parameters for 3-*N*-acetyl amifampridine from Part 2 are not shown). Based on plasma PK profiles with multiple dosing, exposure to the 3-*N*-acetyl amifampridine metabolite was higher than amifampridine in both rapid and slow acetylators, as observed with single-dose administration (Fig. 3). The 3-*N*-acetyl amifampridine/amifampridine exposure ratios from multiple dosing were higher for rapid acetylators with mean AUC_0-24h_ ratios of 59 for rapid acetylators (on Days 1 and 3) versus 5.2–5.7 for slow acetylators (over Days 1 and 3).

A gradual and modest same-day increase in mean AUC_0-4h_ was observed for the 3-*N*-acetyl amifampridine metabolite in both slow (1.56- to 2.00-fold) and rapid acetylators (1.43- to 1.74-fold), which is likely due to the longer *t*_½_ of the metabolite compared to amifampridine. The accumulation of 3-*N*-acetyl amifampridine between Days 1 through 4 was low to negligible in fast (1.00- to 1.31-fold) and slow (1.06- to 1.41-fold) acetylators.

### Safety

**Figure 4:**
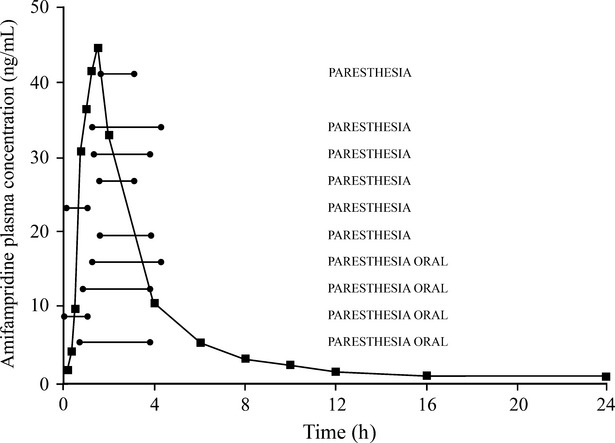
Adverse events versus plasma concentrations. The PK profile represents all plasma concentrations combined as a geometric mean for the *N* = 6 slow acetylator subjects receiving 20 mg doses, in single-dose study Part 1, who reported the most common AEs (paresthesias). The ten horizontal lines represent both the duration (horizontal length) and mean plasma concentration (vertical height equivalent to the mean concentration on the “Y” axis concentration) for the individual ten paresthesia AEs experienced by the six slow acetylator subjects at the 20 mg dose. A geometric mean plasma concentration-time profile is provided rather than six individual plasma concentration-time PK profile plots. Extrapolation to the left and right, until intersection with the plasma concentration-time curve, indicates the concentration ranges over which the paresthesias begin and end, respectively.

Among 12 subjects participating in Part 1, there were 28 treatment-emergent adverse events (TEAEs) which were considered to be related to the study drug by the Medical Investigator and that occurred in slow acetylators only. The most frequently reported drug-related TEAEs were nervous system disorders consisting of general paresthesias (*N* = 11) reported by five subjects and oral paresthesia (*N* = 9) reported by six subjects receiving 20 and 30 mg amifampridine doses in the slow acetylator group (data not shown). All other drug-related TEAEs had an occurrence of 1–2 reports (in 1 patient each) in the slow acetylator group. The geometric mean amifampridine plasma concentration-time profiles for the slow acetylator population, given 20 mg amifampridine in Part 1 (*N* = 6), is summarized in Figure 4 in combination with the duration of the 10 reported paresthesia TEAEs at this dose. All drug-related TEAEs were mild in severity and resolved without sequelae. Together, the data show that the drug-related TEAEs of paresthesia were generally reported when amifampridine showed steep plasma concentration range changes around the maximum and ended when concentrations had steeply decreased, indicating a concentration-dependent nature for these TEAEs.

In the multiple dose study Part 2, drug-related TEAEs by phenotype (*N *=* *51) are summarized in Table 4**.** Similar to study Part 1, the overall number of drug-related TEAEs reported by slow acetylators (*N *=* *45) was greater than those reported by fast acetylators (*N *=* *6) and these were reported by six of seven slow (86%) versus three of seven fast (43%) subjects, respectively. The most frequently reported TEAEs were paresthesia and GI disorders, which were also drug concentration-dependent. All drug-related TEAEs were mild in severity and resolved without sequelae.

**Table 4:** Amifampridine-related treatment-emergent adverse events by phenotype: study part 2, multiple dose (20 mg, QID)

System organ class	Group 1		Group 2		Total	
Preferred term^1^	Fast (*N *=* *2)	Slow (*N *=* *2)	Fast (*N *=* *5)	Slow (*N *=* *5)	Fast (*N *=* *7)	Slow (*N *=* *7)
Total occurrences/patients (%)	4/1 (50)	11/2 (100)	2/2 (40)	34/4 (80)	6/3 (43)	45/6 (86)
Gastrointestinal disorders						
Abdominal discomfort	–	–	–	1/1 (20)	–	1/1 (14)
Nausea	–	–	1/1 (20)	–	1/1 (14)	–
Nervous system disorders						
Dizziness	–	–	–	1/1 (20)	–	1/1 (14)
Paresthesia	2/1 (50)	5/2 (100)	–	10/4 (80)	2/1 (14)	15/6 (86)
Paresthesia oral	2/1 (50)	6/2 (100)	1/1 (20)	22/4 (80)	3/2 (29)	28/6 (86)

Data are expressed as the number (%) of adverse event occurrence/patients.

1 MedDRA Version 13.1.

## Discussion

The genetically polymorphic NAT2 enzyme is responsible for the metabolism and clearance of amifampridine by forming the 3-*N*-acetyl metabolite. Using phenotyping and genotyping methods, we differentiated subjects into a predefined range of slow and fast acetylator phenotypes and evaluated the effect of individual acetylation capacity on the PK profile of amifampridine. Following the administration of single and repeated doses of amifampridine, subjects with the fast acetylator phenotype displayed consistently lower amifampridine *C*_max_, AUC, and a shorter *t*_1/2_ than subjects with the slow acetylator phenotype_._ These results appear to explain the wide interpatient variability in the PK parameters observed in previous amifampridine studies (unpublished data on file, BioMarin). We found excellent (100%) agreement between the phenotype and NAT2 genotype classification of acetylator status in this study. This agreement is also consistent with prior work (Cascorbi et al. 1995) and suggests that both methods may be clinically useful. It is known that the NAT1 enzyme is polymorphic and has overlapping substrate specificity with NAT2, which could contribute to both the overall metabolic phenotype observed for amifampridine in humans and to the observed variability in PK parameters observed within a phenotype. In vitro experimentation with recombinant expressed human NAT1 and NAT2 enzymes indicate that NAT1 can metabolize amifampridine and that NAT2 has an eightfold preferential metabolic rate over NAT1 (Haroldsen et al. 2012). From a therapeutic perspective, the use of NAT2 genotyping may enhance the clinical benefits of amifampridine phosphate in patients with LEMS by minimizing the risk of AEs.

While the results of the present study shed new light on the metabolism of amifampridine, the effects of NAT2 polymorphism on the metabolism of other therapeutic agents (Meyer and Zanger 1997) such as procainamide (Okumura et al. 1997), hydralazine (Schwartz and Turner 2004), and dapsone (Relling 1989) has previously been described. Patients with slow acetylator phenotypes are more susceptible to adverse reactions from several commonly used drugs such as sulfasalazine (Tanigawara et al. 2002), sulfamethoxazole (Soejima et al. 2007), and isoniazid (Singh et al. 2009).

Diminished drug metabolism capacity especially impacts drugs with a narrow therapeutic index. With metabolic-based clearance by *N*-acetylation, amifampridine exposure will be lower to varying degrees for a patient with one or more fast NAT alleles compared to someone without any fast alleles. For example, patients with slow acetylator phenotypes are at risk for isoniazid-induced hepatotoxicity (Ben Mahmoud et al. 2011; Sotsuka et al. 2011). Isoniazid metabolism among patients with one high-activity NAT2 allele may be 50% higher than in patients with no such alleles, but lower than patients with two such alleles (Kinzig-Schippers et al. 2005), requiring corresponding changes in dosing to achieve the desired therapeutic effect. The safety and efficacy of other drugs with a narrow therapeutic index that undergo metabolism *via* acetylation may also benefit from pharmacogenomic studies (Meisel 2002; Zhou et al. 2008).

Although higher exposure to the *N*-acetyl metabolite was observed in the present study, the metabolite levels produced are not expected to result in potassium channel blockade in vivo. The metabolite was determined in in vitro studies to have an IC_50_ > 3000 *μ*mol/L when tested in a panel of six separately cloned human potassium channels (hKv1.1, hKv1.2, hKv1.3, hKv1.4, hKv1.5, and hKv1.7) transiently expressed in mammalian cells (unpublished data on file, BioMarin). This contrasts with a mean single-dose plasma *C*_max_ range of 0.286–1.25 *μ*mol/L in slow and 0.545–2.32 *μ*mol/L in fast acetylators that is three orders of magnitude below the IC_50_ for K^+^ channel blockade.

The clinical significance of different acetylation rates in patients receiving amifampridine for the treatment of LEMS remains to be determined. Currently, the therapeutic dose of amifampridine phosphate is tailored to individual patient needs by gradual dose titration to a maximum dose of 60–80 mg per day or until dose-limiting AEs intervene (Bever et al. 1990; Firdapse® Tablets Prescribing Information 2013). While gradual dose titration reduces safety risks in slow acetylators, fast acetylators may require the maximum recommended dose to achieve higher systemic exposure. While NAT2 genotype analysis provides important prediction for expected PKs and drug exposure, as shown in this report, several other variables may also contribute to the amifampridine metabolism, clearance, and drug exposure (Parkinson et al. 2010). For instance, the absorption rate of amifampridine in the gut may vary between individuals. Second, during the first hepatic pass, not only NAT2 but also NAT1 isoenzyme may provide additional metabolic elimination pathway. Further, amifampridine and its metabolite 3-*N*-acetyl amifampridine may interact with renal transporters affecting the rate of renal excretion in the major route of elimination. Nevertheless, NAT2-driven metabolism of arylamines, including amifampridine, in the liver is the major pathway effecting eventual PK properties of the arylamine class of drugs. NAT2 is also highly expressed in the gut and may contribute to extrahepatic metabolism and elimination of the drug (Parkinson et al. 2010).

Drug-related AEs were considered mild in severity and there were no serious AEs (SAE) reported among the subjects in this study. The most frequently reported drug-related AEs were peripheral and oral paresthesias, which have been reported in previous clinical amifampridine studies (McEvoy et al. 1989; Bever et al. 1990; Sanders et al. 2000; Oh et al. 2009; Wirtz et al. 2009); however, SAEs including seizures have been associated with excessive doses of amifampridine or coadministration with cholinergic agonists such as pyridostigmine (Lindquist and Stangel 2011). Future studies may determine whether serious amifampridine-related AEs are more frequently associated with slow acetylator status.

## Conclusion

Variations in polymorphic NAT corresponding to fast and slow acetylator phenotypes and genotypes significantly affect the PK and safety profile of amifampridine. Single doses of amifampridine demonstrated dose linear PK in both phenotypes, but fast acetylators displayed lower *C*_max_, AUC, and shorter *t*_1/2_ than slow acetylators. Although no drug accumulation or time-dependent changes in PK parameters occurred, slow acetylators reported over 80% more drug-related AEs than fast acetylators. Clinically, identifying NAT phenotypes or genotypes of patients treated with amifampridine phosphate has the potential to improve the drug-related safety and efficacy.

## Abbreviations

1U: 1-methylurate, 3
1X: 1-methylxanthine
3,4-DAP: 3,4-diaminopyridine
AAMU: acetylamino-6-amino-3-methyluracil
AE: adverse event
AFMU: 5-acetylamino-6-formylamino-3-methyl uracil
HPLC: high-performance liquid chromatography
LEMS: Lambert–Eaton Myasthenic Syndrome
NAT: aryl N-acetyltransferase
NAT1: arylamine N-acetyltransferase type 1
NAT2: arylamine N-acetyltransferase type 2
PK: pharmacokinetic
SAE: serious AEs

## Supporting Information

**Table S1**. Summary of human NAT2 gene polymorphisms. **Table S2.** Summary of mean pharmacokinetic parameter ranges for amifampridine in slow and fast acetylators following multiple doses.
