# Supplementary material for: Genetic variation in aryl N-acetyltransferase results in significant differences in the pharmacokinetic and safety profiles of amifampridine (3,4-diaminopyridine) phosphate
Source: Pharmacol Res Perspect. 2014 Dec 9;3(1):e00099. doi: 10.1002/prp2.99 (PMC4317230; doi:10.1002/prp2.99)
Supplement: Supplementary file 1 [file prp20003-e00099-sd1.docx]

**Supplementary Tables**

| **Table S1**. **Summary of Human NAT2 Gene Polymorphisms** | | |
| --- | --- | --- |
| **T341C** | **C282T** | **Acetylator Phenotype** |
| Wildtype | Wildtype | Fast |
| Wildtype | Heterozygote | Fast |
| Wildtype | Mutant | Slow |
| Heterozygote | Wildtype | Fast |
| Heterozygote | Heterozygote | Slow |
| Heterozygote | Mutant | Slow |
| Mutant | Wildtype | Slow |
| Mutant | Heterozygote | Slow |
| Mutant | Mutant | Slow |
| Mutant = variant allele on each chromosome; Heterozygote= mutant allele on one chromosome; Wildtype = no mutant allele | | |

| **Table S2. Summary of Mean Pharmacokinetic Parameter Ranges for Amifampridine in Slow and Fast Acetylators Following Multiple Doses** | | | | | | | | | | | | | | |
| --- | --- | --- | --- | --- | --- | --- | --- | --- | --- | --- | --- | --- | --- | --- |
|  | | **AUC_0-4h_**  **(ng-h/mL)** | | **AUC_0-24h_**  **(ng-h/mL)** | **Cmax (ng/mL)** | | **Tmax (hr)** | | **t ½ (hr)** | | **Vz/F (L)** | | **CL/F (L/hr)** | |
|  | | Low | High |  | Low | High | Low | High | Low | High | Low | High | Low | High |
| **Fast Acetylators: Day 1 (QID Dosing)** | | | | | | | | | | | | | | |
| Day 1 | Mean (SD) | 22.5 (9.81) | 24.1 (11.1) | 97.9 (39.7) | 13.3 (7.58) | 24.4 (17.6) | 0.58 (0.17) | 1.07 (0.45) | 1.12 (0.27) | 1.49 (0.59) | 1334 (386) | 1732 (847) | 757 (225) | 898 (421) |
|  | CV (%) | 43.6 | 45.9 | 40.6 | 57.2 | 72.2 | 28.7 | 42.0 | 24.4 | 39.7 | 28.9 | 48.9 | 29.7 | 46.9 |
|  | Dose | 2 | 3 | 1-4 | 2 | 1 | 3 | 2 | 1 | 4 | 1 | 4 | 2 | 1 |
| **Slow Acetylators: Day 1 (QID Dosing)** | | | | | | | | | | | | | | |
| Day 1 | Mean (SD) | 115 (22.4) | 152 (17.9) | 630  (112) | 67.1 (20.9) | 83.7 (35.1) | 0.86 (0.20) | 1.29 (1.32) | 1.39 (0.26) | 2.52 (0.94) | 269 (52.5) | 338 (73.7) | 101 (35.3) | 152 (29.7) |
|  | CV (%) | 19.6 | 11.8 | 17.8 | 31.1 | 42.0 | 22.9 | 103 | 18.5 | 37.1 | 19.5 | 21.8 | 34.9 | 19.5 |
|  | Dose | 1 | 2 | 1-4 | 4 | 2 | 3 | 4 | 1 | 4 | 3 | 4 | 4 | 1 |
| **Fast Acetylators: Day 3 (QID Dosing)** | | | | | | | | | | | | | | |
| Day3 | Mean SD | 24.9 (12.2) | 28.5 (17.3) | 111  (62.1) | 16.2 (5.61) | 24.0 (18.1) | 0.53 (0.21) | 0.90 (0.65) | 1.07 (0.21) | 1.82 (0.64) | 1112 (310) | 2200 (1753) | 607 (248) | 973 (695) |
|  | CV(%) | 49.1 | 60.9 | 55.9 | 34.6 | 75.6 | 39.6 | 72.4 | 19.6 | 35.1 | 27.8 | 79.7 | 40.8 | 71.5 |
|  | Dose | 1 | 2 | 1-4 | 4 | 1 | 3 | 2 | 1 | 4 | 2 | 3 | 2 | 1 |
| **Slow Acetylators: Day 3 (QID Dosing)** | | | | | | | | | | | | | | |
| Day 3 | Mean SD | 130 (28.8) | 168 (21.6) | 701  (74.3) | 80.9 (20.4) | 97.1 (34.2) | 0.60 (0.22) | 1.00 (0.40) | 1.60 (0.24) | 3.56 (0.49) | 241 (34.0) | 376 (75.7) | 73.2  (10.0) | 124 (19.3) |
|  | CV (%) | 22.3 | 12.9 | 10.6 | 25.2 | 35.3 | 37.3 | 39.5 | 14.9 | 13.8 | 14.1 | 20.1 | 13.7 | 15.6 |
|  | Dose | 1 | 4 | 1-4 | 3 | 4 | 4 | 1 | 1 | 4 | 3 | 4 | 4 | 1 |
| **Fast Acetylators: Day 4 (Single Dose)** | | | | | | | | | | | | | | |
| Day 4 | Mean SD | 22.6  (10.0) | | 25.9 (12.9) | 13.6  (6.60) | | 0.90  (0.45) | | 1.95  (0.72) | | 1774  (388) | | 673  (195) | |
|  | CV (%) | 44.1 | | 49.9 | 48.4 | | 50.5 | | 37.0 | | 21.9 | | 29.0 | |
| **Slow Acetylators: Day 4 (Single Dose)** | | | | | | | | | | | | | | |
| Day 4 | Mean SD | 133  (21.9) | | 186  (32.5) | 72.5  (43.9) | | 1.20  (0.60) | | 3.24  (1.03) | | 486  (91.3) | | 108  (17.6) | |
|  | CV (%) | 16.5 | | 17.5 | 60.6 | | 49.7 | | 31.9 | | 18.8 | | 16.3 | |
